# The association between resting functional connectivity and dispositional optimism

**DOI:** 10.1371/journal.pone.0180334

**Published:** 2017-07-12

**Authors:** Qian Ran, Junyi Yang, Wenjing Yang, Dongtao Wei, Jiang Qiu, Dong Zhang

**Affiliations:** 1 Department of Radiology, Xin Qiao Hospital, The Third Military Medical University, Sha Pingba, Chongqing, China; 2 Key laboratory of cognition and personality (SWU), Ministry of Education, Chongqing, China; 3 School of psychology, Southwest University, Chongqing, China; Banner Alzheimer's Institute, UNITED STATES

## Abstract

Dispositional optimism is an individual characteristic that plays an important role in human experience. Optimists are people who tend to hold positive expectations for their future. Previous studies have focused on the neural basis of optimism, such as task response neural activity and brain structure volume. However, the functional connectivity between brain regions of the dispositional optimists are poorly understood. Previous study suggested that the ventromedial prefrontal cortex (vmPFC) are associated with individual differences in dispositional optimism, but it is unclear whether there are other brain regions that combine with the vmPFC to contribute to dispositional optimism. Thus, the present study used the resting-state functional connectivity (RSFC) approach and set the vmPFC as the seed region to examine if differences in functional brain connectivity between the vmPFC and other brain regions would be associated with individual differences in dispositional optimism. The results found that dispositional optimism was significantly positively correlated with the strength of the RSFC between vmPFC and middle temporal gyrus (mTG) and negativly correlated with RSFC between vmPFC and inferior frontal gyrus (IFG). These findings may be suggested that mTG and IFG which associated with emotion processes and emotion regulation also play an important role in the dispositional optimism.

## Introduction

Dispositional optimism is an important product of human evolution and an individual characteristic that plays a substantial role in human experience [1, 2]. Dispositional optimism can be described as the expectation of positive outcomes. For example, optimistic individuals are confident that they will attain their goals as their expectation [3–5]. Although extreme optimism can be harmful as it can promote an underestimation of risk and poor planning [6, 7], moderate optimism can motivate adaptive behavior in the present towards a future goal [7], and is beneficial to both physical and psychological wellbeing [8–10]. Optimists tend to have lower self-reported depressive symptoms [11], whereas pessimists report more negative expectations of the future [12–14]. Additionally, optimists were observed to have a lower risk of cardiovascular disease compared with pessimists [11, 15], and to be associated with lower incidence rate of complications and better recovery after surgery[16]. Furthermore, optimists are likely to benefit in the social domain [17]. For instance, there is an association between expecting positive outcomes and having broader social networks [18].

Although optimism is highly significantly linked with various aspects of human behavior, the most reliable connection exists between optimism and negative affect[19]. Optimistic individuals have more positive attitudes in daily life, and tend to make active efforts to rectify bad moods whilst having higher self-esteem than pessimistics [4, 20]. When faced with undesirable information, optimistic individuals tend to use reappraisal as an emotional regulation strategy and are therefore more likely to report better moods than less optimistic individuals [2, 20]. Those with lower level of optimism expect bad outcomes, which results in negative feelings such as anxiety, anger, sadness and even despair [17]. It can be inferred, based on this information, that subjects with higher dispositional optimism may regulate their emotions better and experience more positive feelings. In contrast, pessimistic subjects experience more negative feelings, which may be associated with more emotional regulation processing.

The ventromedial prefrontal cortex (vmPFC) is a key region of the resting-state default mode network (DMN), which is important for self-referential function including processing of internal and external cues, recollecting the past, and projecting into the future [21]. Neuroimaging studies have consistently identified the vmPFC involved with self-referential processing [22, 23] and regulation of emotion [24, 25]. Specifically, the vmPFC is related with personal subjective value for social rewards [26], personal significance for self-related processing [23], and is involved in inhibition of amygdala activity to regulate the negative mood [27–29]. In contrast, dysfunction of the vmPFC is thought to be critical in a number of brain disorders, most notably post-traumatic stress disorder (PTBS) [30], depression [31] and dysregulation related to chronic stress [25]. The vmPFC is thought to play an important role in representing conceptual information relevant for survival and integrating concepts into affective behavioral and physiological responses [25].

Previous fMRI studies have shown that increased activity within the vmPFC gives response to positive relative to negative potential future events [7, 32], suggesting that the function of the vmPFC is associated with individual differences in dispositional optimism. However, it remains unclear whether there are other brain regions that contribute in combination with the vmPFC to dispositional optimism.

Communication between different brain regions could be crucial in complex cognitive processes [33]. Examination of resting-state functional connectivity, which reflects temporal correlations between blood oxygen level-dependent signals in different brain regions during rest, can indicate directly or indirectly functional relations between brain regions [34, 35]. Previous studies demonstrated that the RSFC for the region of interest (ROI) had a high degree of test-retest reliability [36, 37].

Neural substrates of optimism have been explored in recent functional magnetic resonance imaging (fMRI) studies, which indicated that ventral medial prefrontal cortex (vmPFC), superior and inferior frontal gyrus (SFG and IFG), anterior cingulate cortex, orbitofrontal cortex, amygdala, and posterior cingulate cortex are recruited when people make positive evaluations of their skills, personality, and future [2, 7, 38]. Therefore, we hypothesized that dispositional optimism might be associated with RSFC between the vmPFC and these optimism-related brain regions.

## Materials and methods

### Participants

In total, 330 healthy individuals (mean age: 19.97 ± 1.27 years; males: 144) from Southwest University, China, participated voluntarily in this study as part of our ongoing project to examine the association between brain imaging, creativity and mental health. All participants were right-handed, with no history of neurological or psychiatric problems.

All participants were university students or alumni from the local community of Southwest University in China. Participants were screened prior to scanning to confirm healthy development by a self-report questionnaire, and therefore, those participants who had a history of psychiatric or neurological disorders, received mental health treatment or had taken psychiatric medications were excluded. All participants provided written informed consent prior to the study. The Brain Imaging Center Institutional Review Board of Southwest University approved this study and its procedures in accordance with the standards of the Declaration of Helsinki (1991).

### Assessment of dispositional optimism

The current study used the Chinese version of LOT-R (Life Orientation Test-Revised) [39, 40] to assess participants’ levels of dispositional optimism [2, 12]. The LOT-R includes 10 items that evaluate generalized expectancies for either positive or negative outcomes. Ratings are made on a 5-point Likert-type scale from 1 (‘I disagree a lot’) to 5 (‘I agree a lot’). Participants were advised to be as accurate and honest as possible and to avoid allowing their answers to previous questions influence their answers to later questions. Only 6 of the 10 LOT-R items are used to derive an optimism score. Four of the items are filler items and are not used in the scoring. Respondents were advised to be as accurate and honest as possible throughout, and to avoid letting their answers to one question influence their answers to other questions. They were told explicitly that there was no right or wrong answers. Negatively worded items (i.e., items 3, 7, and 9) are reverse-scored. All scores of the 6 items are then added together to compute an overall optimism score [39]. Total scores ranged from 6 to 30, with higher scores indicating increased levels of dispositional optimism. The original LOT-R has a high degree of internal consistency (α = 0.80), and substantial research supports its reliability and validity [41]. The Chinese version of LOT-R was reported with weaker internal reliability but still exhibited decent convergent and discriminant validity [40]. However, in present study sample, the internal reliability of the LOT-R is low (α = 0.47) which may be limited the generalization of the results.

### Self-rating negative mood

We used the Self-Rating Depression Scale (SDS) and Self-Rating Anxiety Scale (SAS) to measure participants’ level of depression and anxiety mood. SDS is a self-report measure of depression consisting of 20 items, with a four-point scale ranging from a ‘little of the time’ (1) to ‘most of the time’ (4). Of the 20 items, 10 are worded positively and 10 are worded negatively. The former 10 items are reversed items. The sum score of the 20 items is raw score and the standard score is the raw score multiplied by 1.25. The higher score indicates the higher level of depression. The SDS has good validity and the reliability [42].

SAS is a self-report measure of anxiety consisting of 20 items, with a four-point scale ranging from ‘a little of the time’ (1) to ‘most of the time’ (4). Of the 20 items, 15 are worded positively and 5 are worded negatively. The sum score of the 20 items is raw score and the standard score is the raw score multiplied by 1.25. The higher score indicates the higher level of anxiety. The SAS has good validity and reliability [43, 44].

### Imaging data acquisition

All functional images were obtained from a 3-T Siemens Magnetom Trio scanner (Siemens Medical, Erlangen, Germany). The whole-brain resting-state functional images were acquired using gradient-echo planar imaging (EPI) sequences under the following parameters: slices = 32, repetition time (TR) / echo time (TE) = 2000 / 30 ms, flip angle = 90°, field of view (FOV) = 220 mm × 220 mm, thickness = 3 mm, slice gap = 1 mm, matrix = 64 × 64, resulting in a voxel with 3.4 × 3.4 ×4 mm^3^. The total scanning time is 8 minutes 8 seconds.

### Preprocessing of imaging data

The resting-state image data were processed using both the data processing assistant for resting state software (DPARSF) [45] and the REST toolkit [46]. Both tools were based on the SPM8 software package. The first 10 volumes of the functional images were discarded to account for signal equilibrium and participants’ adaptation to their immediate environment. The remaining 232 images were preprocessed, which included slice timing, head motion correction and spatial normalization to a standard template. The time courses for various co-variates (global signal, white matter, cerebrospinal fluid, and motion parameters for head movement) were extracted and regressed out to cancel out the potential impact of physiological artifacts. We utilized the Friston 24-parameter model to regress out head motion effects from the realigned data. This was based on recent reports that higher-order models demonstrate benefits in reducing head micro-movements [47, 48].

We also addressed the residual effects of motion in group analyses by including the mean frame-wise displacement (FD) derived with Jenkinson's relative root mean square algorithm as a nuisance co-variate [48, 49]. The images were then resampled to 3-mm cubic voxels before spatial smoothing was applied (6 mm FWHM). The smoothed data were linearly de-trended and filtered using a band pass filter (0.01–0.08 Hz) to eliminate low frequency fluctuations. The preprocessing steps of functional connectivity followed the standard protocol reported by Yan and Zang [50–52].

### Functional connectivity analysis

Functional connectivity was examined using a ROI seed method. The seed regions were defined as spheres with a 6-mm radius in the left vmPFC (−3, 42, −21) and right vmPFC (3, 42, −21) as reported in previous studies [53]. To generate the functional connectivity map, the averaged time series was obtained from the ROI and the correlation analysis was conducted between the ROI and the voxel in the whole brain. The correlation coefficient map was then converted into a z-map by Fisher’s r-to-z transformation to improve the normality.

A multiple linear regression analysis was used to identify brain regions in which the RSFC strength with the vmPFC was significantly correlated with the individual dispositional optimism measured by the LOT-R. Previous studies had indicated that some aspects of brain asymmetries interact with gender [54, 55]. Age also has an appreciable effect on brain morphology [56]. Therefore, although the participants’ ages only ranged from 17 to 27 years in the present study, age, sex and mean framewise displacement were entered as co-variates into the regression model to control for possible confounding effects. To increase the power to detect brain functional connectivity within individual difference of optimism, topological false discovery rate (FDR, voxel-corrected p = 0.001 and cluster-corrected p = 0.05) were applied to correct for multiple comparisons in the present study in the whole-brain level [57, 58].

## Results

### Behavioral data

The kurtosis (0.122) and skewness (-0.238) of the LOT-R scores were between -1 and +1, indicating normality of the data [59]. Table 1 shows the mean, range and standard deviation of participants’ age, LOT-R scores, SDS scores and SAS scores (detailed information please see S1 Table). Table 2 shows the distribution of the LOT-R scores. The LOT-R scores were negatively correlated with SDS scores (r = -0.389, P < 0.001) and SAS scores (r = -0.245, P < 0.001), but not correlated with age (P = 0.636, r = −0.026) or sex (t = 1.04, P = 0.292). The results may be suggested that higher optimism individuals has less negative mood in the daily life.

**Table 1:** The mean, range and the standard deviation of age, LOT-R scores, SDS and SAS scores of the participants (N = 330; male = 144).

Measure	Mean	SD	Range
Age	19.97	1.27	17–27
LOT-R scale	21.12	3.18	10–28
SDS scores	43.15	8.43	25–67.5
SAS scores	39.26	7.79	25–82.5

**Table 2:** Distribution of LOT-R scale scores of the participants.

	10–14	15–18	19–22	23–26	27–28
LOT-R scores	9	55	163	86	17

### Correlation of the strength of RSFC with the vmPFC

**Fig 1:**
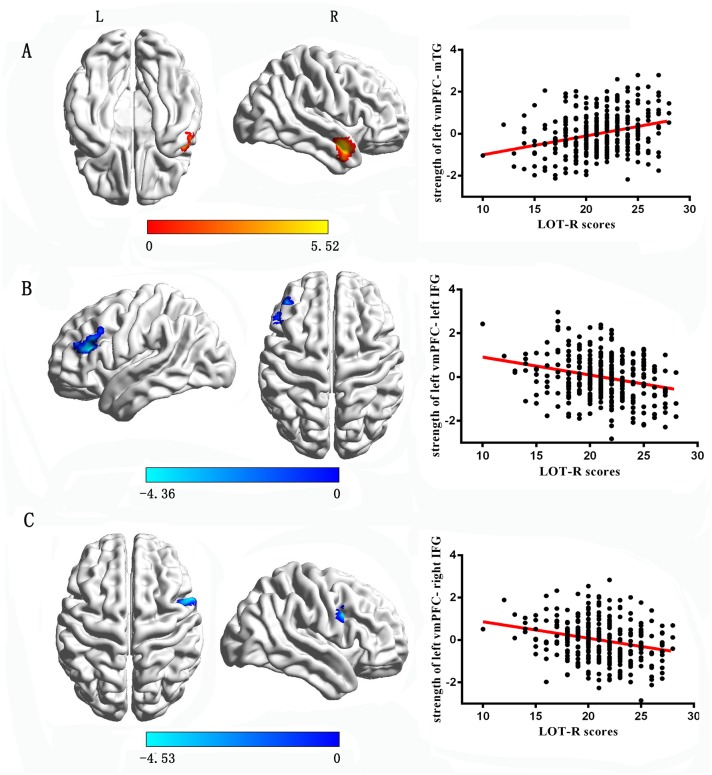
The correlations between optimism and the RSFC with the left vmPFC (with global signal regression). A: The regions and partial correlations scatterplot of positive association of optimism and the strength of RSFC between the left vmPFC and mTG. B: The regions and partial correlations scatterplot of negative association of optimism and the strength of RSFC between the left vmPFC and left IFG. C: The regions and partial correlations scatterplot of negative association of optimism and the strength of RSFC between the left vmPFC and right IFG.

**Fig 2:**
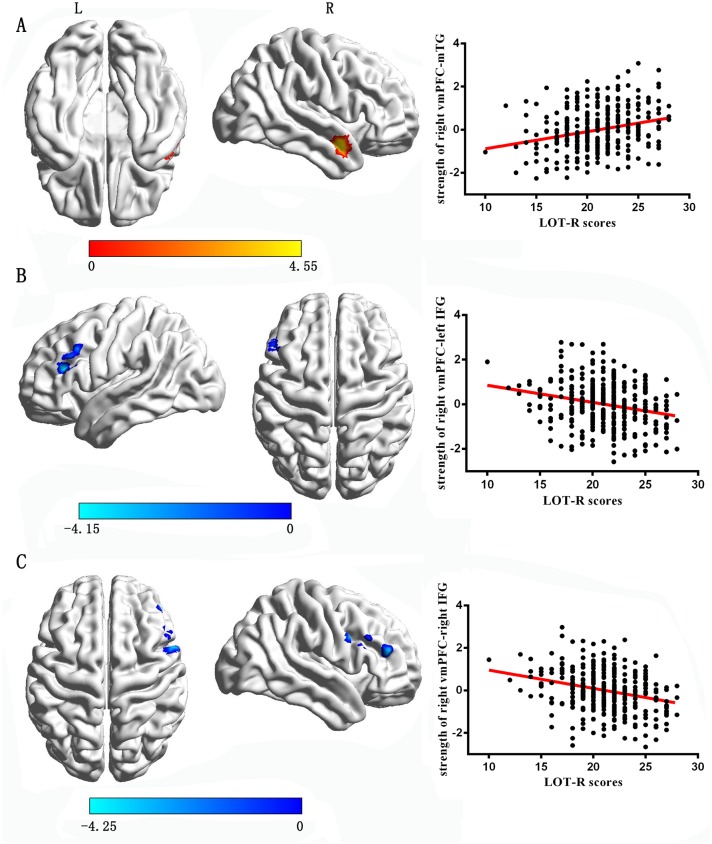
The correlations between optimism and the RSFC with the right vmPFC (with global signal regression). A: The regions and partial correlations scatterplot of positive association of optimism and the strength of RSFC between the right vmPFC and mTG. B: The regions and partial correlations scatterplot of negative association of optimism and the strength of RSFC between the right vmPFC and left IFG. C: The regions and partial correlations scatterplot of negative association of optimism and the strength of RSFC between the right vmPFC and right IFG.

With the left vmPFC as the seed region, multiple regression analysis revealed that the LOT-R score was positively correlated with the strength of RSFC between the left vmPFC and the middle temporal gyrus (x = 51, y = 3, z = -21, cluster size = 107 voxels, t = 5.52, P < 0.05 corrected for topological FDR; see Fig 1A and Table 3), and negatively correlated with the strength of RSFC between the left vmPFC and the bilateral inferior frontal gyrus (IFG, x = -54, y = 27, z = 27, cluster size = 122 voxels, t = -4.36, P < 0.05 corrected for topological FDR, see Fig 1B; x = 60, y = 12, z = 33, cluster size = 69 voxels, t = -4.53, P < 0.05 corrected for topological FDR, see Fig 1C and Table 3). With the right vmPFC as the seed region, the LOT-R score was marginal significantly positive correlated with the strength of RSFC between the left vmPFC and the middle temporal gyrus (x = 51, y = 3, z = -21, cluster size = 107 voxels, t = 4.55, P = 0.055 corrected for topological FDR; see Fig 2A and Table 3), and negatively correlated with the strength of RSFC between the right vmPFC and the bilateral inferior prefrontal gyrus (IFG, x = -54, y = 27, z = 27, cluster size = 56 voxels, t = -4.15, P < 0.05 corrected for topological FDR, see Fig 2B; x = 60, y = 12, z = 33, cluster size = 97 voxels, t = -4.25, P < 0.05 corrected for topological FDR, see Fig 2C and Table 3).

**Table 3:** The results of the functional connectivity.

Brain regions	voxels size	Peak T value	MNI coordinates		
			x	Y	Z
**Left vmPFC**					
mTG	107	5.52	51	2	-21
Left IFG	122	-4.36	-54	27	27
Right IFG	69	-4.53	60	12	33
**Right vmPFC**					
mTG	57	4.55	51	3	-21
Left IFG	56	-4.15	-54	27	27
Right IFG	97	-4.25	60	12	33

Due to the controversial nature of global signal regression, as well as some negative RSFC values in our data, we also conduct a data preprocessing without global signal regression and performed the same multiple regression analysis to examine whether the results remain stable.

**Fig 3:**
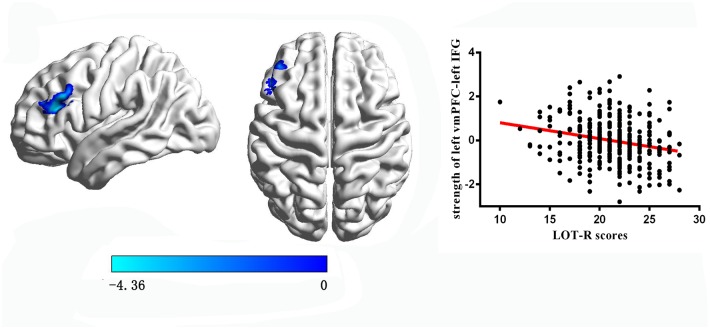
The correlations between optimism and the RSFC with the left vmPFC (without global signal regression). The regions and partial correlations scatterplot of negative association of optimism and the strength of RSFC between the left vmPFC and left IFG.

The results found that the LOT-R score was negatively correlated with the strength of RSFC between the left vmPFC and the left IFG (x = -54, y = 27, z = 30, cluster size = 98 voxels, t = -4.36, P < 0.05 corrected for topological FDR; see Fig 3; detailed information please see S1 File). However, there were no significant correlations with the strength of RSFC between the right vmPFC and other regions within the topological FDR.

## Discussion

This study used resting-state functional magnetic resonance imaging to investigate the relationship between RSFC and dispositional optimism, as measured by the LOT-R. The results showed that individual dispositional optimism was significantly positively correlated with the strength of RSFC between the vmPFC and mTG, and significantly negatively correlated with the strength of RSFC between the vmPFC and bilateral IFG.

The results revealed that there is a positive correlation between dispositional optimism and RSFC between the vmPFC and mTG. Previous studies suggested that the mTG plays a role in emotion processes and emotion regulation [60, 61]. For example, some researchers found that there was greater activity in the mTG when participants viewed sad films compared with neutral films[62]. Therefore, the mTG is an emotion-related brain region [63, 64]. Additionally, some studies suggested that the mTG plays a role in cognitive reappraisal [65–67].

Perhaps the most well-studied strategy to regulate emotion is reappraisal, which involves reinterpreting the meaning of affective stimuli in ways that alter their emotional impact [68]. Optimistic individuals are more positive in their attitude in daily life, make an active effort to rectify bad moods and have higher self-esteem than less optimistic individuals [4, 20]. When faced with undesirable information, optimistic individuals use reappraisal as an emotional regulation strategy and are more likely to report better moods than less optimistic individuals [2, 20]. Taken together, the results suggest that the increased RSFC between the vmPFC and mTG that was linked to a higher level of dispositional optimism might be due to the connections between emotion processes and emotion regulation functions.

There is high evidence suggesting that the IFG is important for flexibly altering beliefs [69–71]. For example, it is important in reversal learning and has been shown to track and integrate information into prior beliefs. Both left and right IFG have been associated with dispositional optimism [2, 72, 73]. Optimistic individuals were worse at tracking undesirable errors in right IFG than those with low scores on optimism[2].

Another study showed that transcranial magnetic stimulation (TMS) of the left IFG increased updating of the unfavorable information[72]. Our analysis found that the RSFC between bilateral vmPFC and bilateral IFG were negatively correlated with dispositional optimism. Since the vmPFC plays an important role in self-referential processing, lower strengh of RSFC between IFG and vmPFC may affect the updating the negative information to the self, which may contribute to higher level of optimism. The IFG also associated cognitive regulation of emotion [74] and various forms of inhibition (such as inhibition of unwanted memories)[75]. Pessimistic individuals by contrast tend to have more negative emotions, which may be associated with more emotional regulation process thus increasing the RSFC between IFG and vmPFC.

We had to notice some limitations in the current study. First, the LOT-R scale has a lower reliability in present study, which may be limited the generalization of these results. Second, because recruiting exclusively right-handed healthy college students, our interpretations might not be generalized to other samples. Finally, only the negative mood of the participants was assessed with a self-report measure, rather than the negative effects.

## Conclusion

This study found that dispositional optimism was significantly positively correlated with the strength of the RSFC between vmPFC and mTG, and negatively correlated with RSFC between vmPFC and IFG. The increased RSFC between the vmPFC and mTG that was linked to a higher level of dispositional optimism might be due to connections between the emotion process and emotion regulation functions. The decreased RSFC between the vmPFC and IFG that was linked to a higher level of dispositional optimism might be due to connections between emotion regulation and self-referential processing.

Although the correlational design of this study does not allow conclusions regarding the causality of the assessed factors, the RSFC pattern we found could inform us about a better understanding of the the neural basis of dispositional optimism and its relationship with emotion regulation and self-referential processing.

## Supporting information

S1 Table This is LOT-R, SAS and SDS score. (XLSX)

S1 File This is fMRI results. (RAR)
